# Association Between 18-FDG Positron Emission Tomography and MRI Biomarkers of Plaque Vulnerability in Patients With Symptomatic Carotid Stenosis

**DOI:** 10.3389/fneur.2021.731744

**Published:** 2021-12-23

**Authors:** Nicola Giannotti, Jonathan McNulty, Shane Foley, John McCabe, Marey Barry, Morgan Crowe, Eamon Dolan, Joseph Harbison, Gillian Horgan, Eoin Kavanagh, Martin O'Connell, Michael Marnane, Sean Murphy, Ciaran Mc Donnell, Martin O'Donohoe, David Williams, Peter J. Kelly

**Affiliations:** ^1^School of Medicine, University College Dublin, Dublin, Ireland; ^2^Neurovascular Unit for Translational and Therapeutics Research, Mater Misericordiae University Hospital, Dublin, Ireland; ^3^Vascular Surgery, St. Vincent's University Hospital, Dublin, Ireland; ^4^Department of Medicine for the Elderly, St. Vincent's University Hospital, Stroke Service, Dublin, Ireland; ^5^Stroke and Hypertension Unit, Connolly Hospital, Dublin, Ireland; ^6^Acute Stroke Service, St. James Hospital Dublin, Trinity College Dublin, Dublin, Ireland; ^7^Health Research Board (HRB) Stroke Clinical Trials Network Ireland, University College Dublin, Dublin, Ireland; ^8^Department of Radiology, Mater Misericordiae University Hospital, Dublin, Ireland; ^9^Department of Vascular Surgery, Mater Misericordiae University Hospital, Dublin, Ireland; ^10^Geriatric Medicine, Beaumont Hospital and Royal College Surgeons Ireland, Dublin, Ireland

**Keywords:** PET, MRI, atherosclerosis, vulnerable plaque biomarker, carotid, plaque inflammation, plaque segmentation

## Abstract

**Purpose:** Pathologic studies suggest that unstable plaque morphology and inflammation are associated with cerebrovascular events. ^18^F-fluorodeoxyglucose positron emission tomography (^18^FDG-PET) is a validated technique for non-invasive imaging of inflammation-related plaque metabolism, and MRI can identify morphologic features of plaque instability. The aim of this study was to investigate the association of selected imaging characteristics of plaque vulnerability measured with MRI and PET in patients with symptomatic carotid stenosis.

**Methods:** Patients from the BIOVASC study were selected based on the following inclusion criteria: (1) age ≥ 50 years; (2) recent (<30 days) ischaemic stroke (modified Rankin scale ≤3) or motor/speech/vision TIA; (3) ipsilateral internal carotid artery stenosis (≥5 0% lumen-narrowing); (4) carotid PET/CTA and MRI completed. Semi-automated plaque analysis of MRI images was performed to quantify morphologic features of plaque instability. PET images were co-registered with CTA and inflammation-related metabolism expressed as maximum standardised uptake value (SUV_max_).

**Results:** Twenty-five patients met inclusion criteria (72% men, mean age 65 years). MRI-measured plaque volume was greater in men (1,708–1,286 mm^3^, *p* = 0.03), patients who qualified with stroke (1,856–1,440 mm^3^, *p* = 0.05), and non-statin users (1,325–1,797 mm^3^, *p* = 0.03). SUV_max_ was associated with MRI-measured plaque lipid-rich necrotic core (LRNC) in the corresponding axial slice (*r*_*s*_ = 0.64, p < 0.001) and was inversely associated with whole-plaque fibrous cap thickness (*r*_*s*_ = −0.4, *p* = 0.02) and calcium volume (*r*_*s*_ = −0.4, *p* = 0.03).

**Conclusion:** This study demonstrated novel correlations of non-invasive imaging biomarkers of inflammation-related plaque metabolism with morphological MRI markers of plaque instability. If replicated, our findings may support the application of combined MRI and PET to detect vulnerable plaque in future clinical practise and randomised trials.

## Introduction

Recurrent stroke and coronary events occur in 4–6% of stroke survivors each year, despite guideline-based treatment (1). New approaches to address this residual vascular risk are urgently needed. The current assessment of carotid atherosclerotic lesions is based on luminal stenosis measurements and surface defects using *in vivo* imaging techniques including digital angiography, CT, MRI, and ultrasonography (2). However, histopathologic studies suggested that morphological plaque characteristics of instability and inflammation may be associated with an increased risk for cerebrovascular events (3, 4). The identification of carotid plaque containing a large lipid-rich necrotic core (LRNC) with intraplaque haemorrhage (IPH) and thin or ruptured fibrous cap (FC) may assist physicians to identify symptomatic or asymptomatic patients at higher risk for future stroke.

MRI is a validated technique for characterising luminal stenosis, plaque volume, and composition. Positron emission tomography (PET) using ^18^F-fluorodeoxyglucose (FDG) has been validated for non-invasive imaging of inflammation-related plaque metabolism (5, 6). Almost no data exist on the association between plaque inflammation imaged with PET and biomarkers of unstable plaque imaged with MRI in patients with recently symptomatic carotid atherosclerosis. Therefore, using an imaging dataset of symptomatic patients recruited as part of a larger, multi-centre prospective cohort study Biomarkers Imaging Vulnerable Atherosclerosis in Symptomatic Carotid disease (BIOVASC), we aimed to investigate the association between plaque inflammation measured as SUV_max_ on ^18^FDG-PET and MRI biomarkers of plaque vulnerability in patients with symptomatic carotid stenosis.

## Methods

### Eligibility Criteria

Pre-specified inclusion criteria of the BIOVASC study were: (1) age ≥ 50 years; (2) presentation to medical attention with recent (<30 days) non-severe ischaemic stroke (modified Rankin scale [MRS] ≤ 3) or motor/speech/vision transient ischaemic attack (TIA); (3) ipsilateral internal carotid artery (ICA) stenosis (≥ 50% lumen-narrowing) on admission Doppler ultrasound, magnetic resonance angiogram (MRA) or CT angiography (CTA) done for clinical care; (4) PET/CTA completed. The main exclusion criteria were: (1) possible haemodynamic stroke/TIA due to carotid near-occlusion; (2) contraindication to contrast-enhanced CT; (3) unsuitability for carotid PET/CTA, MRI, or research participation. For the current study, we selected patients who had high-resolution carotid wall MRI completed no later than 7-days from PET/CTA.

The study was approved by relevant Ethics Committees and patients gave informed consent. All procedures performed in studies involving human participants were in accordance with the ethical standards of the institutional and/or national research committee and with the 1964 Helsinki declaration and its later amendments or comparable ethical standards.

### Image Acquisition

#### PET/CT

F-fluorodeoxyglucose (^18^FDG) PET/CT was performed using a Siemens Biograph 16 scanner (Siemens, Erlangen, Germany) after a minimum 6 h fast. Blood glucose level was verified for each patient and if above 11 mmol/L the PET/CT scan was not performed. Then, 320 megabecquerel (MBq) of ^18^FDG was administered 2 h prior to image acquisition. The uptake phase was standardised with the patient resting. PET images were acquired in three-dimensional (3D) mode in two bed positions for 10 min each. Slice thickness of 3 mm and a 256 × 256 matrix were used. PET emission mode images were acquired and reconstructed by applying the OSEM2D4i24s algorithm and XZY Gauss 2 convolution kernel (Siemens Healthineers, Erlangen, Germany). A low-dose CT scan for attenuation correction was performed using the same scanner directly after PET; in addition, where the administration of a contrast agent (Omnipaque 350, GE Healthcare, Milwaukee, USA) was not contraindicated (serum creatinine level >1.5 mg/dl or estimated glomerular filtration rate < 60 ml/min) a diagnostic carotid CTA was performed using bolus tracking. The pre-monitoring slice was set at the aortic arch, and a circular region of interest (ROI) was drawn distant from any vessel calcification. CT images (1 mm slice thickness, with contrast enhancement) were acquired from the aortic arch to the skull base to identify carotid arteries and jugular veins. CTA parameters were 120 kVp, 104 mAs, 512 × 512 matrix, pitch 0.6 and 1-mm CT slice reconstructions following the acquisition. A smooth reconstruction kernel was used (b30f).

#### MRI

Carotid arteries were scanned from the common carotid artery to a point distal to the internal carotid artery stenosis where the vessel wall is parallel. Patients were scanned with Siemens Avanto 1.5T MR (Siemens Healthineers, Erlangen, Germany) with a dedicated phased-array surface neck coil (Machnet BV, Netherlands). The carotid bifurcation of the symptomatic side was identified with the MR localiser. Following this, 3D time-of-flight (TOF) MR Angiography (MRA) and axial T1w, T2w, proton density-weighted (PD), and T1w post-contrast sequences were acquired along the length of the vessel wall. Double inversion-recovery (IR) sequences were used to allow blood signal nulling with cardiac synchronisation to reduce wall motion. T1w sequences were acquired prior to and post-injection of 20 mls of Gadobutrol (Gadovist, AG Bayer, Berlin, Germany).

Scanning parameters: field of view 256 × 256 mm; 2 mm slice thickness and 0.2 slice interval; time to repetition (TR)/time to echo (TE) were 978/12, 1,880/62, and 1,880/12 for T1, PD, and T2, respectively. Voxel size 0.5 × 0.5 × 2 mm and NEX of 1. Moreover, a 40° flip-angle and a short TE (<7 ms) were used in the TOF sequence to maximise the contrast between stationary tissues and flowing blood. The total scan time was 23.2 min per patient.

### Image Analysis

Quality assurance (QA) cheques were performed on PET/CT and MRI prior to commencing the study to ensure that the scanners were performing according to recommended international standards. Further QA cheques were performed on the MRI and PET/CT imaging datasets before commencing the image analysis.

All images were centrally analysed by a single trained reader, including re-measurement of CTA images to confirm the degree of stenosis according to the NASCET criteria (7). Intra-rater reliability assessment showed excellent agreement between carotid CTA measurements taken at different time-points (intraclass correlation α = 0.814, *p* < 0.001) (8). Following semi-automated co-registration of PET and CT images (Osirix, Pixmeo, Geneva), carotid ^18^F-FDG activity in 10 regions of interest (ROI) defined relative to the slice of maximal stenosis was quantified using standardised uptake values (SUV g/ml = measured uptake (MBq/ml) / injected dose (MBq) per patient weight [g]).

The whole plaque was defined as the volume of the carotid artery corresponding to 10 ROIs drawn on 10 1 mm CTA slices (1 cm length in total) using the point of maximal stenosis as the mid-point of the whole plaque segment. The whole-plaque SUV represents the SUV averaged across the 10 ROIs. Moreover, we defined the single hottest slice (SHS) as the axial slice with maximal SUV uptake (SUV_max_) and most diseased segment (MDS) as SHS plus the adjacent proximal and distal axial slices, corresponding to a 3 mm long plaque segment (9).

Following a semi-automatic co-registration of MRI sequences (T1-weighted, T2-weighted, TOF, and proton density-weighted) and lumen-plaque boundaries segmentation, carotid plaque morphological features were semi-automatically measured with MRI-Plaque View 2 (VPDiagnostic, Seattle, WA, USA). MRI measures included plaque volume (mm^3^), plaque thickness (mm^2^), LRNC volume (mm^3^), intra-plaque haemorrhage (IPH) volume (mm^3^), fibrous cap (FC) thickness (mm), and normalised wall index (NWI). The NWI is defined as plaque wall area/(lumen + wall area).

**Figure 1:**
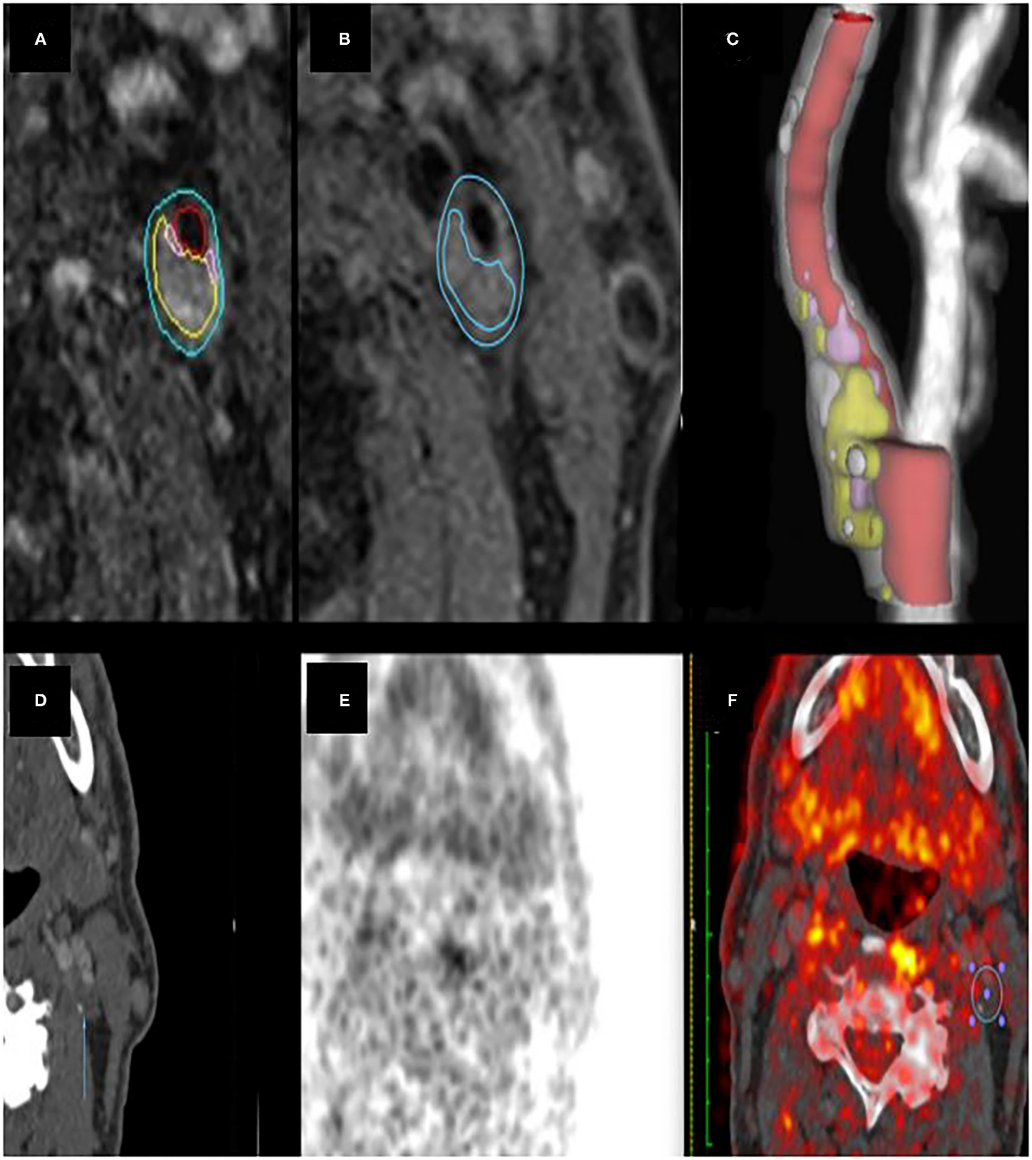
**(A)** carotid MRI semi-automatic segmentation of lumen and vessel wall. **(B)** semi-automatic plaque characterisation of an LRNC area. **(C)** ICA plaque 3D Volume Rendering. **(D–F)** CT and PET images of the same plaque area. The ROI **(F)** shows where SUV_max_ was measured.

Because inflammation may be non-uniformly distributed across carotid plaques, the association of SHS-SUV_max_ with MRI morphological features was first analysed and compared to the corresponding axial MRI slice (matching slice analysis) (Figure 1). The analysis was then repeated, comparing the MDS-SUV_max_ to MRI morphological features across the entire measured plaque (whole-plaque analysis).

Between-group characteristics were compared using pre-specified analyses which included *t*-tests, Mann-Whitney, or χ^2^ tests. Non-parametric associations between continuous variables were analysed using Spearman's correlation test. Linear regression analysis was performed to investigate the strength of the association between plaque inflammation and clinical characteristics.

## Results

### Clinical Characteristics

The study group consisted of 25 patients, among which 40% (10 patients) with severe ICA stenosis (Table 1). Furthermore, 10 patients presented with stroke (40%), while 15 patients (60%) with a transient ischaemic attack (TIA). One patient had stroke recurrence and four had TIA recurrence within 90 days. NWI was the only MRI metric that was significantly greater in patients with recurrent events (93 vs. 87.7, *p* = 0.05). MRI whole-plaque volume was greater in men (1,707.7 vs. 1,285.9, *p* = 0.03), non-statin users (1,325.3 vs. 1,797.3, *p* = 0.03), patients with stroke as index event (1,856 vs. 1,439.7, *p* = 0.05). LRNC volume was greater in men (121.1 vs. 39.3, *p* = 0.03) and mean plaque calcium volume was greater in patients with hypertension (209.3 vs. 64.6, *p* < 0.01) (Table 2). NWI was associated with plaque LRNC volume (rho = 0.49, *p* = 0.01).

**Table 1:** Patient demographics.

**Characteristic**	**Patients**
Total number	25
Age, years (mean, range)	65 (55–86)
Hypertension, *n* (%)	16 (64.0%)
Current Smoking, *n* (%)	13 (52%)
Statin at presentation, *n* (%)	11 (41%)
Aspirin at presentation, *n* (%)	11 (41%)
Diabetes mellitus, *n* (%)	3 (11%)
Type of index cerebrovascular event	
Stroke, *n* (%)	10 (40.0%)
TIA, *n* (%)	15 (60.0%)
Stenosis category (NASCET)	
Moderate 50–69%	15 (60%)
Severe > 70%	10 (40.0%)

**Table 2:** Distribution of MRI plaque features and clinical characteristics.

	**Mean plaque volume (mm^3^)**	***p***	**Mean FC thickness (mm)**	***p***	**Mean IPH volume (mm^3^)**	***p***	**Mean LRNC volume (mm^3^)**	***p***	**Mean calcium volume (mm^3^)**	***p***	**Mean NWI**	***p***	**Mean SUV**	**p**	**SHS-SUV**	***p***	**MDS-SUV**	***p***
**Gender**																		
- Male	1,707.7		1.3		23.1		121.1		169.9		90		1.77		2.88		2.80	
- Female	1,285.9	0.03	1.0	0.18	10.1	0.81	39.3	0.03	124.5	0.85	85	0.1	1.93	0.39	2.81	0.79	2.75	0.85
**Hypertension**																		
- Yes	1,678.1		1.3		19.7		106.4		209.3		88.7		1.74		2.68		2.63	
- No	1,432.3	0.27	0.9	0.91	20.1	0.18	84.9	0.42	64.6	0.002	88.4	0.95	1.95	0.19	3.18	0.06	3.05	0.1
**Current smoking**																		
- Yes	1,558.3		1.2		26.8		117.4		136		89.9		1.72		2.99		2.91	
- No	1,623.5	0.76	1.2	0.99	12.3	0.36	78.4	0.23	180.2	0.53	87.4	0.25	1.90	0.26	2.71	0.29	2.65	0.3
**Statin at presentation**																		
- Yes	1,325.3		1.2		15.3		75.1		146.7		88.1		1.75		2.62		2.59	
- No	1,797.3	0.03	1.2	0.54	25.4	0.95	117.1	0.71	43.5	0.41	89.3	0.61	1.86	0.46	3.04	0.11	2.94	0.16
**Diabetes mellitus**																		
- Yes	1,256.3		0.9		23.4		101.9		97.6		88.3		1.63		2.53		2.53	
- No	1,635.1	0.26	1.2	0.86	19.4	0.92	98.2	0.8	165.4	0.93	88.8	0.93	1.84	0.39	2.91	0.35	2.81	0.45
**Index event**																		
- Stroke	1,856		1.0		28.1		137.3		200.1		86.5		1.74		2.65		2.60	
- TIA	1,439.7	0.05	1.3	0.15	15.2	0.33	76.9	0.21	133.1	0.25	89.9	0.14	1.86	0.45	2.98	0.22	2.89	0.27
**Stenosis category**																		
- Moderate 50–69%	1,778.3		1.2		22.5		104.1		189.5		87.5		1.77		2.77		2.69	
- Severe > 70%	1,349.5	0.04	1.2	0.82	17.6	0.69	90.6	0.74	108.8	0.35	90.3	0.22	1.88	0.5	2.99	0.42	2.91	0.38
**Stroke recurrence**																		
- Yes	1,494.2		1.5		5.56		105.4		85.1		92.9		1.79		2.72		2.65	
- No	1,613.5	0.66	1.1	0.63	23.4	0.52	97.0	0.59	175.3	0.15	87.7	0.05	1.91	0.54	3.42	0.14	3.30	0.13

#### Association of ^18^F-FDG PET Plaque Inflammation With Plaque MRI Features

On analysis of corresponding axial slices, SUV_max_ SHS was associated with greater LRNC volume (rho = 0.64, *p* = 0.001), but not other MRI features of plaque instability (Table 3).

**Table 3:** Correlation between plaque FDG uptake and plaque MRI features (Spearman's correlation coefficient).

	**Matching slice analysis**		**Plaque analysis**	
	**SUV_**max**_-SHS**	***p***	**SUV_**max**_–MDS**	***p***
Calcium volume	−0.17	0.41	−0.43	0.03
FC thickness	−0.68	0.74	−0.44	0.02
IPH volume	−0.96	0.65	0.33	0.11
LRNC vol	0.64	0.001	0.09	0.64
Plaque volume	−0.15	0.49	−0.13	0.55
NWI	−0.30	0.14	−0.15	0.40

*For analysis of FDG uptake and MRI features in matching slices, FDG is expressed as SUV_max_ in the corresponding axial slice. For analysis of FDG uptake and MRI features across the plaque, FDG is expressed as SUV_max_ in the MDS*.

On analysis of whole-plaque MRI features, SUV_max_ MDS was inversely associated with plaque calcium volume (rho = −0.43, *p* = 0.03) and fibrous cap thickness (rho = −0.44, *p* = 0.02) (Table 3). SUV_max_ MDS showed a weak trend towards association with serum LDL-cholesterol (*r*_*s*_ = 0.34, *p* = 0.09).

**Figure 2:**
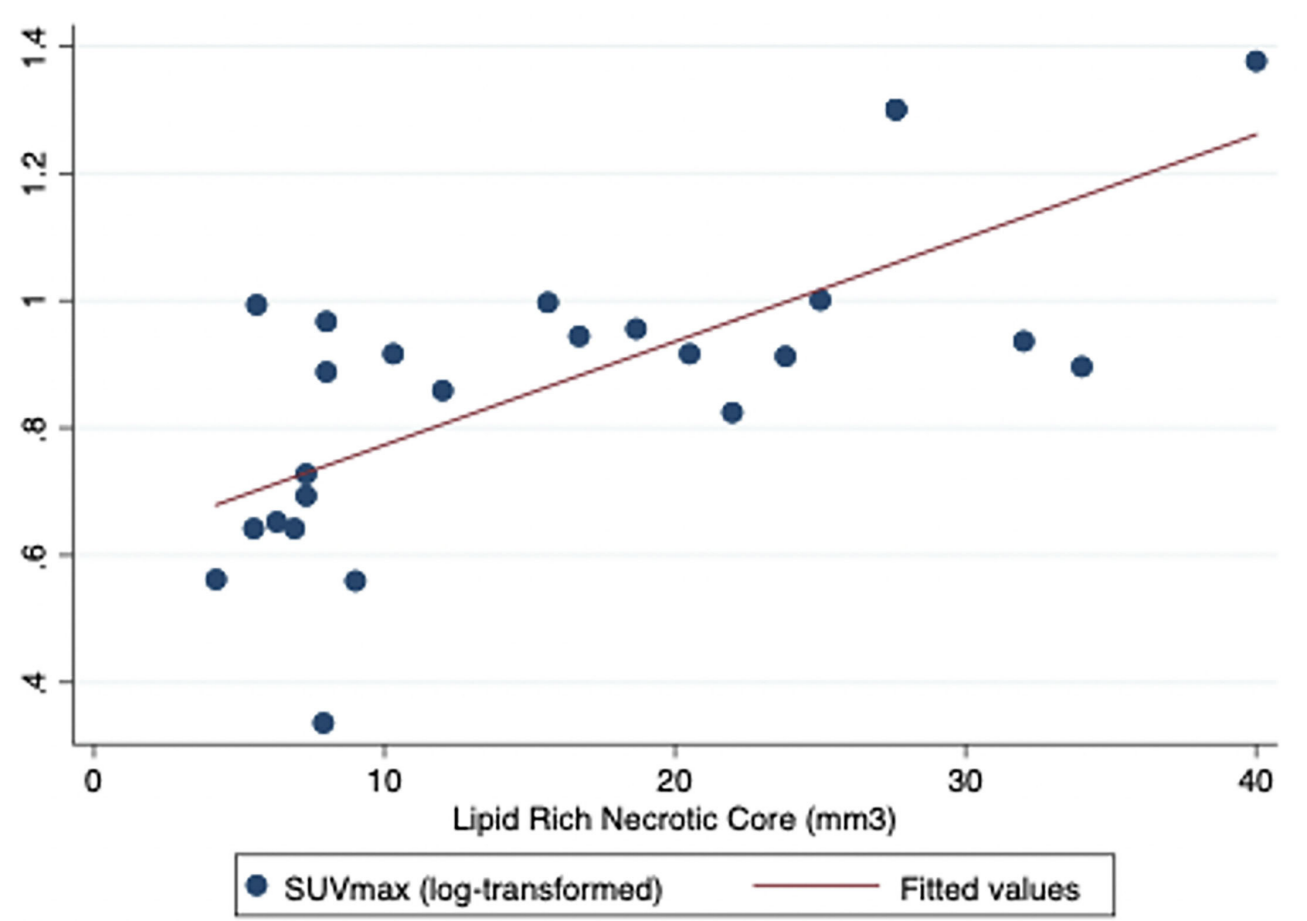
Regression analysis figure for LRNC and SUV max (SHS).

On linear regression analysis, plaque FDG uptake (measured as log-transformed SUV_max_ SHS to meet normality assumptions of regression analysis) was associated with LRNC area at the corresponding slice (*R*^2^.5, *p* = 0.001, coefficient.016, standard error.003) suggesting that approximately half the variance in plaque SUV uptake was explained by LNRC area (Figure 2). No other associations between FDG uptake and MRI morphology were observed on linear regression analysis of corresponding slices.

## Discussion

In recently symptomatic patients with stroke or TIA, we investigated the relationship between morphological MRI biomarkers of unstable carotid plaque and inflammation-related plaque metabolism measured by ^18^FDG-PET/CTA. We found positive associations between plaque inflammation and lipid-rich core volume in corresponding axial slices, and inverse (negative) correlations between inflammation and markers of plaque stability (plaque calcification and fibrous cap volume).

Few previous studies have investigated the combined use of carotid wall MRI and molecular imaging with PET/CT in patients with atherosclerosis. In non-stroke subjects who underwent serial whole-body combined FDG-PET/MRI, FDG uptake was associated with the number and volume of atherosclerotic plaques, and with plaque lipid content and positive remodelling (10). In 61 patients with carotid stenosis and recent symptoms, plaque SUV_max_ was associated with serum LDL cholesterol, total cholesterol, and triglycerides, and inversely associated with HDL cholesterol (11). However, plaque lipid content was not measured in this study. In 18 patients with cryptogenic stroke and non-stenosing carotid atheroma, the presence and size of MRI-measured lipid core in ipsilateral carotid plaque were associated with FDG uptake (12). Similar findings were reported in a Chinese MRI/PET study of asymptomatic patients with non-stenosing carotid plaque (13).

Few data exist relating other MRI morphological features with FDG uptake. Inverse associations were observed between FDG uptake and ipsilateral carotid plaque fibrous cap thickness in patients with cryptogenic stroke and non-stenosing plaque, and in asymptomatic Chinese patients thicker caps and calcification were associated with lower FDG uptake (12, 13). We found no association between FDG uptake and IPH, unlike 2 earlier studies that reported positive associations (12, 14). Two other studies reported associations between plaque neovascularisation measured by dynamic contrast-enhanced MRI and plaque inflammation measured by PET (15, 16).

The main strength of our study is the novelty of its findings, as very little data exist on combined PET and MRI carotid plaque imaging datasets in recently symptomatic patients. Both unstable plaque morphology and inflammation are validated markers that identify patients at the highest stroke risk. If validated in further studies, our results may support a rationale for use of combined PET/MRI plaque imaging for improved risk stratification of patients in future randomised trials for carotid revascularisation or may improve the cost-effective targeting of next-generation anti-atherosclerotic medications towards high-risk patients (17).

The main limitation is the limited sample size, which may have resulted in insufficient statistical power for some analyses. The sample of data used in this study was collected from patients enrolled in the larger BIOVASC study where carotid symptomatic patients only were recruited. Although the SHS/MDS methods are standard for such studies, we also acknowledge technical limitations for spatial resolution of current PET scanners. Due to limitations of spatial resolution of PET, we cannot exclude the possibility that FDG uptake in the MDS may partially reflect spill-over of signal from adjacent proximal and distal plaque segments (~1–1.5 mm in each direction).

Further studies involving a larger number of participants are needed. We acknowledge that some variability may exist in the matched slices analysis. Although the most optimal slice selection of PET/CT and MRI images was made using defined protocols and the carotid bifurcation as a reference, patient positioning and technical limitations of each imaging modality may introduce variability in the analysis of the two imaging datasets.

### Summary

Although further research is needed, these initial findings suggest that inflammation-related plaque metabolism measured with PET/CT may be associated with morphological MRI biomarkers of plaque vulnerability, suggesting that the use of both PET and MRI may be a promising approach to assess new anti-atherosclerotic treatments to prevent stroke in patients with carotid stenosis.

## Data Availability Statement

The raw data supporting the conclusions of this article will be made available by the authors, on reasonable request.

## Ethics Statement

The studies involving human participants were reviewed and approved by Mater Misericordiae University Hospital, Dublin, Ireland. Ref 1/378/1131. The patients/participants provided their written informed consent to participate in this study.

## Author Contributions

NG, JM, SF, and MB: contributed to study design, data acquisition, data analysis, and manuscript preparation. MC, ED, JH, EK, MO'C, MM, SM, CD, MO'D, and DW: contributed to the study design and manuscript preparation. GH: contributed to the study design, data acquisition, and manuscript preparation. PK is the principal investigator of the BIOVASC study, planned the study design and contributed to data acquisition, data analysis, outcome adjudication, and manuscript preparation. All authors have read and approved the final manuscript.

## Funding

This study was partially supported and funded by the Health Research Board (Ireland) (CSA/201227) and the Irish Institute of Radiography and Radiation Therapy (IIRRT). PK: HRB Clinician Scientist and Clinical Trials Network Awards, and Irish Heart Foundation. NG: Irish Institute of Radiography and Radiation Therapy. Funders had no input into the data analysis or manuscript preparation.

## Conflict of Interest

The authors declare that the research was conducted in the absence of any commercial or financial relationships that could be construed as a potential conflict of interest.

## Publisher's Note

All claims expressed in this article are solely those of the authors and do not necessarily represent those of their affiliated organizations, or those of the publisher, the editors and the reviewers. Any product that may be evaluated in this article, or claim that may be made by its manufacturer, is not guaranteed or endorsed by the publisher.

## Abbreviations

LRNC: Lipid rich necrotic core
MDS: Most diseased segment
SHS: Single hottest slice.
